# Data-Driven Method for Efficient Characterization of Rare Event Probabilities in Biochemical Systems

**DOI:** 10.1007/s11538-018-0509-0

**Published:** 2018-09-17

**Authors:** Min K. Roh

**Affiliations:** Institute for Disease Modeling, 3150 139th Ave SE, Bellevue, WA 98005 USA

**Keywords:** Stochastic simulation, Rare event probability estimation, SSA, dwSSA, Gillespie algorithm, Importance sampling

## Abstract

As mathematical models and computational tools become more sophisticated and powerful to accurately depict system dynamics, numerical methods that were previously considered computationally impractical started being utilized for large-scale simulations. Methods that characterize a rare event in biochemical systems are part of such phenomenon, as many of them are computationally expensive and require high-performance computing. In this paper, we introduce an enhanced version of the doubly weighted stochastic simulation algorithm (dwSSA) (Daigle et al. in J Chem Phys 134:044110, 2011), called dwSSA$^{++}$, that significantly improves the speed of convergence to the rare event of interest when the conventional multilevel cross-entropy method in dwSSA is either unable to converge or converges very slowly. This achievement is enabled by a novel polynomial leaping method that uses past data to detect slow convergence and attempts to push the system toward the rare event. We demonstrate the performance of dwSSA$^{++}$ on two systems—a susceptible–infectious–recovered–susceptible disease dynamics model and a yeast polarization model—and compare its computational efficiency to that of dwSSA.

## Introduction

When Gillespie (1976, 1977) introduced the stochastic simulation algorithm (SSA), its use was deemed purely academic as computers were not powerful enough to support SSA simulations except for toy models. SSA is an exact numerical method in that its trajectories can be used to construct the chemical master equation (CME) as the number of simulations reach infinity. Every reaction is simulated explicitly (reaction time and index) until the final simulation time is reached for each trajectory. This can be computationally infeasible for a large system or even for a small system with many reaction firings. However, as computer processors became more affordable and powerful, increasing number of researchers started using the SSA to model a biological system and gained useful insight from numerical simulations. The dramatic increase in the usage can be seen by the number of citations SSA received; Gillespie’s paper (1977) was cited less than 100 times annually until 2003, and the number of annual citations spiked up to over 500 after 2007 (https://scholar.google.com/citations?user=QwXwK6UAAAAJ#).

With the popularity of SSA came new algorithms derived from it. Some were developed to increase the computational efficiency of the exact method (Gibson and Bruck 2000; Ramaswamy et al. 2009; Slepoy et al. 2008), while others featured faster computation at the expense of accuracy (Cao et al. 2007; Ben Hammouda et al. 2017; Tian and Burrage 2004; Auger et al. 2006; Gillespie 2001; Munsky and Khammash 2006). Specialized methods stemmed from SSA as well when researchers realized various scientific communities shared an interest in specific system behavior or characteristics, such as multiple timescale simulation (Chevalier and El-Samad 2009; Ball et al. 2006; Goutsias 2005; Cao et al. 2004, 2005), model reduction (Kang and Kurtz 2013; Gillespie et al. 2009), steady-state dynamics (Mauch and Stalzer 2010; Grima et al. 2012), and rare event characterization (Donovan et al. 2013; Zelnik et al. 2015; Xu and Cai 2011; Kuwahara and Mura 2008; Gillespie et al. 2009; Roh et al. 2010, 2011). The last area, field of rare event characterization, is relatively new because of the exceptionally high computational requirements associated with estimating a rare event probability. In order to obtain an accurate estimate, an exact method must be used. Accuracy lost from using an approximate method is likely to be much greater than the magnitude of the rare event probability. Moreover, variance of the estimate decreases slowly, proportional to the square root of the total number of simulations. Despite these hurdles, many important events in biology, chemistry, and epidemiology are rare and stochastic by nature. Examples of a significant rare event include mutation of a normal cell into a cancerous cell (Wang et al. 2014; Luebeck and Moolgavkar 2002; Moolgavkar and Knudson 1981), phage $\lambda$ (Cao et al. 2010; Arkin et al. 1998), development of multidrug-resistant bacteria (Nikaido 2009; Maisonneuve et al. 2013), and resurgence of a disease Watts et al. (2005).

Development of the weighted stochastic simulation algorithm (wSSA) by Kuwahara and Mura (2008) alleviated some of the computational tolls by using importance sampling (IS) in the reaction selection process. In wSSA bias introduced by IS parameters is recorded at each reaction selection step and used at the end of the simulation to obtain an unbiased estimate of the rare event probability. Doing so does not affect the accuracy of wSSA, and with a good choice of IS parameters, a significant reduction in variance can be achieved. However, there are two major drawbacks with the wSSA. First is that the method does not provide any means to assess the accuracy of the resulting estimate. It is well known that a bad choice of IS parameters can yield an estimate whose variance is higher than that of an unbiased estimator. This problem was solved when Gillespie et al. (2009) demonstrated that running sum of trajectory weights can be used to compute the uncertainty of the final estimate without affecting the time complexity of wSSA. Second drawback of wSSA is that it did not provide a principled way to choose a good set of IS parameters. Having to guess the value of each IS parameter, one for every reaction is unreasonable even for a modeler who has a considerable insight into the system, especially in the presence of nonlinear reactions. This predicament was addressed by Daigle et al. (2011) with doubly weighted SSA (dwSSA), where both the time to the next reaction and the reaction index are biased. Significance of double weighting (biasing) is that its mathematical form of a trajectory weight can be used to compute a closed-form solution for the optimal IS parameters that minimize cross-entropy, which is used as a proxy to minimum variance. Calculating variance involves, except for a few simple toy models, computation of higher moments, which in turn depend on higher moments. Being able to obtain a closed-form solution is critical for computational efficiency and accuracy, and dwSSA provides an automated and principled way to compute good IS parameters that yield a low-variance estimate. In order to achieve this, Daigle et al. modified and incorporated a *multilevel* version of the cross-entropy (CE) method by Rubinstein and his colleagues (Rubinstein and Kroese 2004; Rubinstein 1997) into the SSA.

While dwSSA offers automatic selection of good IS parameters, its performance highly depends on the convergence rate of the multilevel CE method that computes optimal IS parameters. If the system exhibits low stochasticity, it is likely that dwSSA converges very slowly to the rare event. The worst-case scenario is that the multilevel CE method does not converge and is unable to return IS parameters. Since having a good set of IS parameters is necessary for obtaining a low-variance estimate, failure in the multilevel CE method is detrimental to the performance of dwSSA. In this paper we introduce dwSSA++ that contains a novel and improved method for computing optimal importance sampling parameters when the system is unable to reach the rare event with sufficient speed. In Sect. 2, we review the doubly weighted stochastic simulation algorithm and present the polynomial leaping method that is used to improve the speed of convergence. Pseudo-algorithms are provided in addition to the MATLAB code (https://github.com/minroh/dwSSA_pp) for ease of understanding. We then apply the dwSSA and the dwSSA++ to a susceptible–infectious–recovered–susceptible (SIRS) model to compare their computational efficiency and accuracy in Sect. 3. Finally, we summarize our contributions in Sect. 4.

## Method

### Stochastic Simulation Algorithm and Stochastic Chemical Kinetics

We focus on a well-stirred stochastic system with *N* species $$\{S_1, \ldots , S_N\}$$, who interact through any of *M* reaction channels $$\{R_1, \ldots , R_M\}$$ to change its population in discrete values. The state of the system at time *t* is denoted by $$\mathbf {X}(t) \equiv (X_1(t), \ldots , X_N(t))$$, where $$X_i(t)$$ corresponds to the number of molecules of $$S_i$$ at time *t*. Probability that reaction $$R_j$$ fires in the interval $$[t, t+\mathrm {d}t)$$ is given by its propensity function $$a_j(\mathbf {x}) \equiv a_j(\mathbf {X}(t)), $$$$ \; j \in {1, \ldots , M}$$, where $$\mathrm {d}t$$ is an infinitesimal time increment. The sum of all *M* propensity functions is denoted $$a_0(\mathbf {x})$$.

The SSA simulates time evolution of $\mathbf {x}$ by generating a sequence of samples on two random variables: $\tau$, time elapsing between the current and the next reaction firings; and $j'$, index of the reaction fired at time $t + \tau$. First random variable $\tau$ is exponentially distributed with mean $1/a_0(\mathbf {x})$, while $j'$ is a categorical random variable where the probability of $R_j$ being chosen as the next reaction is $a_j(\mathbf {x})/a_0(\mathbf {x}), \; j \in \{1,\ldots , M\}$. After $\tau$ and $j'$ are computed, we update the state of the system using a $M \times N$ stoichiometry matrix $\mathbf {V}$, whose *j*th row $\nu _j$ indicates the amount of change in $\mathbf {x}$ due to one $R_j$ reaction firing, i.e., $\mathbf {X}(t + \tau ) = \mathbf {X}(t) + \mathbf {V}_{:,j}'$.

### Doubly Weighted Stochastic Simulation Algorithm

We give a brief description of dwSSA here. Further details can be found in Daigle et al. (2011). The goal of dwSSA is to generate trajectories to characterize the probability of reaching a rare event ${\mathscr {E}}$ by final time $t_f$. Thus, a trajectory is simulated until either $t_f$ is reached or event ${\mathscr {E}}$ is observed at a stopping time ${\mathscr {T}} < t_f$, whichever occurs sooner. The form of rare event probability on which the dwSSA operates is $p(\mathbf {x}_0, {\mathscr {E}}; t_f)$; it is defined as the probability that the system starting at time 0 in state $\mathbf {x}_0$ will first reach rare event ${\mathscr {E}}$ by some time $\le t_f$.

Unlike the wSSA (Kuwahara and Mura 2008) or the swSSA (Roh et al. 2010) that limit importance sampling to reaction selection, dwSSA biases both the time to the next reaction $\tau$ and the next reaction index $j'$. There are two significant advantages of dwSSA over wSSA and swSSA. First, the dwSSA makes possible characterization of rare events in some systems that cannot be achieved with the wSSA or the swSSA; second, the dwSSA offers an automated method for choosing importance sampling parameters, ${\gamma } = [\gamma _1, \ldots , \gamma _M]$, that yield a low-variance estimate. Under dwSSA, probability that the reaction $R_j$ fires in the interval $[t, t+\mathrm {d}t)$ is given by its *predilection* function $b_j(\mathbf {x})$ instead of the propensity function $a_j(\mathbf {x})$, where $b_j \equiv a_j \times \gamma _j, \ b_0 = \sum _{j=1}^{M} b_j(\mathbf x),$ and $\gamma _j \in \mathbb {R}^+$. Using the predilection function, $\tau$ now has a mean $1/b_0(\mathbf {x})$, and $j'$ is categorically distributed with probability $b_j(\mathbf {x})/b_0(\mathbf {x})$. Thus, denoting $N_{{\mathscr {T}}}$ as the total number of reactions that fire in the interval $[0,{\mathscr {T}}]$, the probability of a single dwSSA trajectory $\mathbf {J} \equiv (\tau _1, j'_1, \ldots , \tau _{N_{{\mathscr {T}}}}, j'_{N_{{\mathscr {T}}}})$ takes the form

1 $$\begin{aligned} \mathrm {P}_{dwSSA}(\mathbf {J})= & {} \prod _{i=1}^{N_{{\mathscr {T}}}} \left[ b_0(\mathbf {X}(t_i)) e^{-b_0(\mathbf {X}(t_i)) \tau _i} d \tau _i \times \frac{b_{j'_i}(\mathbf {X}(t_i))}{b_0(\mathbf {X}(t_i))} \right] \nonumber \\= & {} \prod _{i=1}^{N_{{\mathscr {T}}}} \left[ b_{j'_i}(\mathbf {X}(t_i)) e^{-b_0(\mathbf {X}(t_i)) \tau _i} d \tau _i \right] , \end{aligned}$$

where $t_i \equiv \sum _{j=1}^i \tau _j$. This probability is clearly biased for $\gamma \ne \mathbf {1}$. Correction factor for the dwSSA trajectory $\mathbf J$, whose product with the probability in (1) equals the probability of an unbiased SSA trajectory, is

2 $$\begin{aligned} W_{dwSSA}(\mathbf {J})= & {} \prod _{i=1}^{N_{{\mathscr {T}}}} \left[ \frac{a_{j'_i}(\mathbf {X}(t_i)) e^{-a_0(\mathbf {X}(t_i)) \tau _i}}{b_{j'_i}(\mathbf {X}(t_i)) e^{-b_0(\mathbf {X}(t_i)) \tau _i}} \right] \nonumber \\= & {} \prod _{i=1}^{N_{{\mathscr {T}}}} \left[ \exp { \left\{ \left( b_0(\mathbf {X}(t_i)) - a_0(\mathbf {X}(t_i)) \right) \tau _i \right\} } \times (\gamma _{j'_i})^{-1} \right] . \end{aligned}$$

It is trivial to check that the product of (1) and (2) is equal to the unbiased probability of a SSA trajectory $\mathbf {J}$:

3 $$\begin{aligned} \mathrm {P}_{SSA}(\mathbf {J})= & {} \prod _{i=1}^{N_{{\mathscr {T}}}} \left[ a_0(\mathbf {X}(t_i)) e^{-a_0(\mathbf {X}(t_i)) \tau _i} d \tau _i \times \frac{a_{j'_i}(\mathbf {X}(t_i))}{a_0(\mathbf {X}(t_i))} \right] \nonumber \\= & {} \prod _{i=1}^{N_{{\mathscr {T}}}} \left[ a_{j'_i}(\mathbf {X}(t_i)) e^{-a_0(\mathbf {X}(t_i)) \tau _i} d \tau _i \right] . \end{aligned}$$

The Monte Carlo estimate for $p(\mathbf {x}_0, {\mathscr {E}}; t_f)$ using dwSSA is thus

4 $$\begin{aligned} \hat{p}_{dwSSA}(\mathbf {x}_0, {\mathscr {E}}; t) = \frac{1}{K} \sum _{k=1}^K \left[ I_{\{S(\mathbf {J}_k) \cap {\mathscr {E}}\}} W_{dwSSA}(\mathbf {J}_k) \right] , \end{aligned}$$

where $\mathbf {J}_k$ represents the $k{\mathrm {th}}$ dwSSA trajectory and $I_{\{S(\mathbf {J}_k) \cap {\mathscr {E}}\}}$ is 1 if any of the states visited by $\mathbf {J}_k$ (denoted by $S(\mathbf {J}_k)$) includes ${\mathscr {E}}$ and 0 otherwise.

Daigle et al. incorporated a modified version of Rubinstein’s cross-entropy method (Rubinstein 1997; Rubinstein and Kroese 2004) in order to minimize the cross-entropy between the unknown optimal $\gamma ^*$ and its numerical estimate $\hat{{\gamma }}^*$, which is used to compute $W_{dwSSA}(\mathbf {J}_k)$ in (2). Significance of minimizing cross-entropy instead of variance is that the former allows for a closed-form solution for $\hat{{\gamma }}^*$ while the latter does not. Minimizing cross-entropy is equivalent to maximizing the following formula:

5 $$\begin{aligned} \max _{{\gamma }} \left( \sum _{k=1}^K \left[ I_{\{S(\mathbf {J}_k) \cap {\mathscr {E}}\}} \times \ln \mathrm {P}_{dwSSA}(\mathbf {J}_k; {\gamma }) \right] \right) . \end{aligned}$$

For many applications, argument inside (5) is convex function of $\gamma$ (Rubinstein and Kroese 2004). Assuming convexity, we can obtain a closed-form solution by taking partial derivatives with respect to each $\gamma _j$ and setting the right-hand side to $\mathbf {0}$:

6 $$\begin{aligned} \sum _{k=1}^K \left[ I_{\{S(\mathbf {J}_k) \cap {\mathscr {E}}\}} \times \mathop {\nabla }_{{\gamma }} \ln \mathrm {P}_{dwSSA}(\mathbf {J}_k; \hat{{\gamma }}^*) \right] = \mathbf {0} \; . \end{aligned}$$

In rare event simulation, (6) can be problematic as most trajectories will not reach ${\mathscr {E}}$, i.e., $I_{\{S(\mathbf {J}_k) \cap {\mathscr {E}}\}}$ will be 0 for most *k*. Daigle et al. solved this problem by using a *multilevel* version of the cross-entropy method (Rubinstein and Kroese 2004), which takes the system closer to ${\mathscr {E}}$ in an iterative manner using favorable signals obtainable from the current state. Starting with $s=1$ and ${\gamma }^{(0)}=\mathbf {1}$, we define an *intermediate* rare event ${\mathscr {E}}^{(s)}$ as the value closest to ${\mathscr {E}}$ that is reachable by top $\rho$ fraction of all trajectories simulated with ${\gamma }^{(s-1)}$. We note that no computation of $\gamma$ is required in the beginning ($s=1$) as the system starts unbiased, i.e., ${\gamma }^{(s-1)}={\gamma }^{(0)}=\mathbf {1}$. After computing ${\mathscr {E}}^{(s)}$, we compute the following closed-form solution to obtain $\hat{\gamma }_j^{(s)}, \, j \in \{1, \ldots , M\}$:

7 $$\begin{aligned} \hat{\gamma }_j^{(s)} = \frac{{\displaystyle \sum }_k' \left( W_{dwSSA}\left( \mathbf {J}_k^{(s-1)}; \hat{{\gamma }}^{(s-1)}\right) \times n_{kj} \right) }{{\displaystyle \sum }_k' \left( W_{dwSSA}\left( \mathbf {J}_k^{(s-1)}; \hat{{\gamma }}^{(s-1)}\right) \times \sum _{i=1}^{N_{{{\mathscr {T}}}_k}} \left[ a_j\left( \mathbf {X}_k^{(s-1)}(t_{ki})\right) \tau _{ki} \right] \right) } \;, \end{aligned}$$

where $n_{kj}$ is the total number of times $R_j$ fires in the $k{\mathrm {th}}$ dwSSA trajectory. In the above expression, rare event indicator function $I_{\{S(\mathbf {J}_k) \cap {\mathscr {E}}\}}$ in (6) has been replaced by summations $\sum _k'$, where *k* iterates only over trajectories reaching the intermediate rare event ${\mathscr {E}}^{(s)}$. This procedure repeats until the intermediate rare event ${\mathscr {E}}^{(s)}$ either surpasses or reaches ${\mathscr {E}}$. At this time we terminate the multilevel CE method and set $\hat{{\gamma }}^* \equiv \hat{{\gamma }}^{(s)}$. The final step is to obtain an estimate for $p(\mathbf {x}_0, {\mathscr {E}}; t_f)$ using $\hat{{\gamma }}^*$. We note that we cannot derive a closed-form solution for $\hat{\gamma }^*$ using the probability expression for wSSA or swSSA. It is a unique feature of the dwSSA and the sdwSSA (Roh et al. 2011), latter of which also employs double biasing but with state-dependent IS parameters. Both the closed-form solution and automatic determination of importance sampling parameters are needed for the algorithm to be of practical use, especially for systems that contain nonlinear reactions.

The algorithm for estimating $p(\mathbf {x}_0, {\mathscr {E}}; t_f)$ with uncertainty (Gillespie et al. 2009) using $\hat{\gamma }^*$ is as follows:
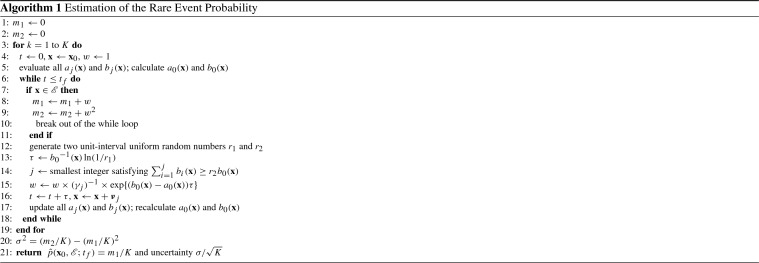


The uncertainty in **step**21 can be used to assess quality of the estimate $\hat{p}(\mathbf {x}_0, {\mathscr {E}}; t_f)$. Denoting the true probability as $p(\mathbf {x}_0, {\mathscr {E}}; t_f)$, the probability that $\left( \hat{p}(\mathbf {x}_0, {\mathscr {E}}; t_f) {-} \sigma /K\right) \le p(\mathbf {x}_0, {\mathscr {E}}; t_f) \le \left( \hat{p}(\mathbf {x}_0, {\mathscr {E}}; t_f)+\sigma /K\right)$ is 68%. Doubling the interval ($2\sigma /K$) raises the confidence level to 95% and tripling to 99.7%. Thus, the smaller the uncertainty is, the tighter the confidence interval will be. If the uncertainty has the same order magnitude as the rare event probability estimate, then there is little to no trust in the value of $\hat{p}_{dwSSA}(\mathbf {x}_0, {\mathscr {E}}; t_f)$, and the user is advised to increase *K* and rerun the algorithm.

### Extrapolation of Biasing Parameters Using Past Simulation Data

The only difference between the dwSSA and dwSSA$^{++}$ lies on how $\hat{{\gamma }}^*$ is computed; given a set of IS parameters, both algorithms compute $\hat{p}(\mathbf {x}_0, {\mathscr {E}}; t_f)$ using Algorithm 1. However, automatic computation of $\hat{{\gamma }}^*$ is the most important component that makes the dwSSA efficient and practical compared to the earlier and related methods (Kuwahara and Mura 2008; Roh et al. 2010, 2011). Without automatic computation of $\hat{{\gamma }}^*$, dwSSA becomes impractical as the user is expected to provide an importance sampling parameter for each reaction in the system. Although a user may be able to guess the general direction of biasing, i.e., encouraging ($\gamma _j > 1$) or discouraging ($\gamma _j < 1$), it is almost impossible for the user to guess values of all IS parameters in the system that can produce a low-variance estimate. In addition, manually tuning IS parameters (Roh et al. 2010, 2011) is not computationally feasible for any large systems. Therefore, except for very simple models, multilevel CE method is expected to run to obtain $\hat{{\gamma }}^*$ prior to starting Algorithm 1.

While the multilevel CE method allows for automatic computation of $\hat{{\gamma }}^*$ that minimizes cross-entropy, its performance largely depends on the speed of convergence to the rare event. For many applications, computational cost of multilevel CE method is negligible compared to the total cost of the simulation since the number of simulations used in multilevel CE method is often orders of magnitude less than that used in Algorithm 1 (Daigle et al. 2011; Roh et al. 2011). It is possible, however, for the computation time in multilevel CE method to dominate the total simulation time. This can happen when the system under study exhibits low stochasticity. If population count is high for all species, there will be little variability among trajectories. Even for a system with small population, IS parameters computed in a prior iteration can bring the system to a strongly stable stochastic equilibrium. In both cases, lack of variability in $\mathbf {x}$ among trajectories is likely to result in an intermediate event that is either close or equal to the system’s average behavior. In fact, it is possible that ${\mathscr {E}}^{(s)}$ is farther from ${\mathscr {E}}$ than ${\mathscr {E}}^{(s-1)}$ . In the worst case, ${\mathscr {E}}^{(s)}$ may never converge to ${\mathscr {E}}$ and no $\hat{{\gamma }}^*$ is computed. For this reason, it is recommended that the user sets a limit of iterations on the multilevel CE method to avoid running *ad infinitum*. For simulations in this paper, we set this number to 20.

In an attempt to address such slow or no-convergence scenarios, we developed a method called polynomial leaping that tries to take the system out of the low-variance region and toward the rare event using past simulation data. When sufficient signal is present, a polynomial interpolant is constructed for each reaction, where the input values are past importance sampling parameter values. Depending on the system’s behavior, polynomial leaping utilizes one of the following two data types as the output variable for the interpolant: number of trajectories that reached ${\mathscr {E}}$ and the value of intermediate rare events from past simulations. Once the output variable is chosen, a low-degree (1 or 2) polynomial interpolant is computed for each reaction, which is then used to extrapolate the next set of IS parameters. The amount of extrapolation depends on the system’s proximity to the rare event as well as on the current speed of convergence. Whenever polynomial leaping method is used to compute the next set of IS parameters, computation of (7) is omitted in the multilevel CE method. Thus, employing polynomial leaping method could not only increase the speed of convergence but also reduce the total number of simulations required to obtain $\hat{p}(\mathbf {x}_0, {\mathscr {E}}; t_f)$. The modified multilevel CE method for dwSSA$^{++}$ with polynomial leaping is described in Algorithm 2.
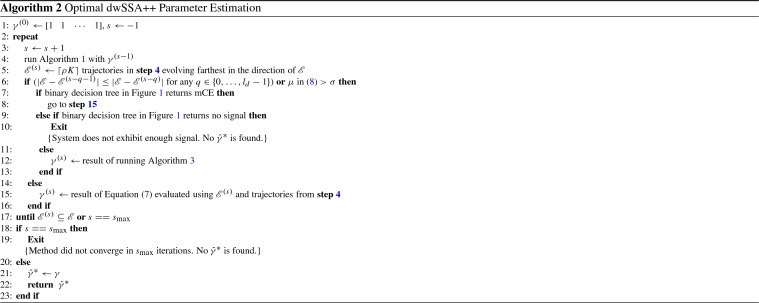


Here, $s_{\text {max}}$ denotes the maximum number of iterations allowed to compute $\hat{{\gamma }}^*$ before declaring the algorithm failed to converge. For examples shown in Sect. 3, we set $s_{\text {max}}$ to 20. The number of past data used to assess convergence rate and form an interpolant is defined as $l_d$, which is shown in **step**6 of Algorithm 2. While $l_d$ can be any integer greater than 1, we recommend that it does not exceed 5. The reason is that increasing the number of data required for interpolation is not likely to increase the quality of the resulting interpolant. If good progress is made toward ${\mathscr {E}}$ with the conventional multilevel CE method, polynomial leaping method will not be called. On the other hand, if the system is converging slowly or not at all, having a large value of $l_d$ delays the initial calling of the polynomial leaping method until at least $l_d$ iterations of multilevel CE method are executed. Starting the polynomial leaping method also implies that past intermediate rare events (IREs) are similar in their values; the same must be true for the importance sampling parameters corresponding to these IREs. Thus, requiring a large number of past data is not expected to significantly increase the quality of resulting interpolant and will delay the system from leaping. For these reasons, we set the default value for $l_d$ to 3.

There are two conditions that can prompt leaping in Algorithm 2 (**step**6). If any one of the two conditions evaluates to be true, then the polynomial leaping method (Algorithm 3) is used to compute ${\gamma }^{(s)}$ instead of **step**15. First condition is true when $l_d$ past intermediate rare events form a non-strictly converging sequence to ${\mathscr {E}}$. This means any stalling or regressing in ${\mathscr {E}}^{(s)}$ values during $l_d$ stages of multilevel CE method will trigger polynomial leaping. Second condition is satisfied if the estimated number of iterations to reach the rare event exceeds a preset threshold $\sigma$, which is set to 5 by default. We obtain the estimated number of iterations, $\mu$, by first computing the speed of progress based on the last two multilevel CE iterations:

8 $$\begin{aligned} \begin{aligned}&h \leftarrow |\mathscr {E}^{(s)} - \mathscr {E}^{(s-1)}|, \text {where } \mathscr {E}^{(s)} \text { and } \mathscr {E}^{(s-1)} \text { are two most recent IREs} \\&\mu = \lceil |{\mathscr {E}} - \mathscr {E}^{(s)} | / h \rceil . \\ \end{aligned} \end{aligned}$$

We note that the above method is based on the relative rate of convergence to $\mathscr {E}$ and does not depend on the absolute distance to the rare event, which depends on the randomly assigned initial reaction rate values.

**Fig. 1:**
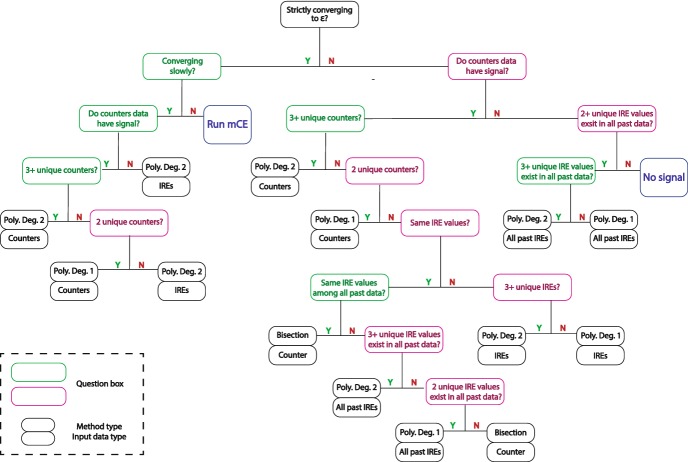
Binary decision tree used in polynomial leaping method. Larger boxes in the figure contain questions used in the decision making process, and its outline color indicates the type of response from its parent node. Box outlined in green is reached if the response to its parent node is positive. Similarly, red box is reached if the response is negative. Leaves of the decision tree represent the type of acceleration method and input data type

In order to determine the leaping eligibility, Algorithm 2 executes a series of diagnostic questions via binary decision tree shown in Fig. 1. The two conditions that trigger leaping correspond to the first node and its left child node, respectively. If neither condition is met, then the multilevel CE method is resumed to determine $\gamma ^{(s)}$ as sufficient progress is being made toward ${\mathscr {E}}$, i.e., $\mu <= \sigma$. This case corresponds to the leaf node with value **Run mCE** in Fig. 1. On the other hand, if the underlying system is neither making a progress toward the rare event nor exhibiting any signal, Algorithm 2 is unable to determine the direction of bias required to reach ${\mathscr {E}}$. While unlikely to occur for most systems, it is theoretically possible. For example, this may happen if the chosen initial state coincides with the system’s strong equilibrium state with very low variance. This case is indicated by leaf node with value **No signal** in Fig. 1. In all other cases, the binary decision tree returns two pieces of information required to initiate leaping (Algorithm 3): method of extrapolation and the type of input data. The method can be either polynomial interpolation or bisection and is decided based on the number of input data available. Bisection is employed only when there is a single eligible data for extrapolation. Polynomial interpolation is used otherwise. Here, we fit a low-degree polynomial according to specifications returned by the binary decision tree. Interpolants constructed by the polynomial leaping method are kept at low degree (1 or 2) since (7) was derived assuming convexity Daigle et al. (2011) and a small number of data, $l_d$, is used to compute the interpolants. We note that the default value for $l_d$ (=3) is set such that it is the minimum number of data required to construct a polynomial interpolant of degree 2. Leaves of the binary decision tree that correspond to polynomial interpolation contain value **Poly.** with its degree (**Deg. 1** or **Deg. 2**). Bisection is indicated by the keyword **Bisection**.

Second piece of information returned by the binary decision tree, type of input data, can be either past counter values or past IRE values. Between these two types, the former is preferred to the latter. Counter data represent the number of trajectories that reached ${\mathscr {E}}$ from past $l_d$ iterations of multilevel CE method. Cardinality of the set of possible values for counters is $\mathbf {card}(\{0,1,\ldots ,\lfloor K \times \rho \rfloor \})$, which is large for commonly chosen values of $K (10^5 \, \text {to} \,10^8)$ and $\rho (10^{-4}\, \text {to} \,10^{-2})$, where smaller value of $\rho$ is associated with larger *K*. Upper limit of this set is $\lfloor K \times \rho \rfloor$, as the multilevel CE method is able to compute $\hat{{\gamma }}^*$ once $(K \times \rho )$ or more number of trajectories reach $\mathscr {E}$. The large range allows the algorithm to easily assess the effect of change in biasing parameter values and compute reliable interpolants. On the other hand, the range of intermediate rare events varies greatly depending on the definition of a rare event for a given system; a wide range of biasing parameters may correspond to the same intermediate rare event. There is one notable advantage in using past IREs, however. We do not need to worry about its existence; unlike counters data, past IRE data is always available regardless of the system’s proximity to $\mathscr {E}$. Unless the system starts in a strong stochastic equilibrium, which is very unlikely given myriad possible combinations of random initial reaction rate values, multilevel CE method will make a progress toward the rare event. The progress does not guarantee any trajectories to reach the rare event, and thus counter data may be 0. Nevertheless, its IRE value will be closer to $\mathscr {E}$ due to the progress. And if the system reaches a strong equilibrium during the simulation and produce $l_d$ IREs with the same value, we can extract more signal by accessing IRE values beyond past $l_d$ iterations. This is the reason queries in the binary decision tree contain checks for all past IREs if the last $l_d$ IRE values are identical. Thus, the order of preferred data type in the algorithm is counters, past $l_d$ IRE values, and all past IRE values.

Once interpolants are constructed, we decide on the value of the output variable $\xi$ that we want the system to produce on the next iteration of Algorithm 2. This value is assigned as the RHS of each *M* interpolant to compute $\gamma _j^{(s)}$. Since $\xi$ is an unobserved value outside the range of past behavior, obtaining $\gamma _j^{(s)}$ is considered extrapolation. We note that computing $\gamma ^{(s)}$ via extrapolation replaces the traditional multilevel CE routine (Algorithm 2, **step**15), saving *K* trajectory simulations per each leaping.

Computing a robust target $\xi$ using past counters is straight forward:

9 $$\begin{aligned} \xi = {\left\{ \begin{array}{ll} \lceil 2 \rho K \rceil &{} \text{ if } \left( \max (\varvec{y}^{\text {past}}) >\frac{\lceil \rho K \rceil }{2}\right) \\ \lceil \rho K \rceil &{} \text {otherwise}, \\ \end{array}\right. } \end{aligned}$$

where $\varvec{y}^{\text {past}}$ represents past counters data. Ideally, we would like to compute IS parameters for ${\mathscr {E}}$, but doing so can result in a large extrapolation error if the current system is far from the rare event. When the maximum of the past counters is greater than half the minimum number of data required to compute $\hat{{\gamma }}^*$, we set the target counter $\xi$ to twice this minimum value ($\lceil 2 \rho K\rceil$) to ensure enough trajectories reach ${\mathscr {E}}$ without over-perturbing the system. When this condition is not met, system is considered *far* from observing the rare event, and we set the target to a more conservative value of $\xi = \lceil \rho K \rceil$.

Using the past IREs for extrapolation is not as straightforward. Speed of convergence can vary greatly depending on the function that defines a rare event. In order to assess the convergence speed, we compute the first order approximation using the amount of progress made by two most recent intermediate rare events. Denoting these two values as $y_1$ and $y_2$, where $y_1$ is the last computed IRE, the target output is computed as following:

10 $$\begin{aligned} \begin{aligned}&h \leftarrow |y_1 - y_2| \\&\mu = |{\mathscr {E}} - y_1| / h \\&\delta = \lceil \min (\mu /2, 3h) \rceil \\&\mathbf{if } \; ({\mathscr {E}} - y_1) < 0 \\&\quad \quad \xi \leftarrow y_1 - \delta \\&\mathbf{else } \\&\quad \quad \xi \leftarrow y_1 + \delta \\&\mathbf{endif } \end{aligned} \end{aligned}$$

In the above equation, the quantity *h* reflects the absolute amount of progress made in IRE from the most recent simulation, and $\mu$ denotes the number of iterations required to reach the rare event assuming the amount convergence per iteration stays at *h*. We then compute the desired amount of progress for the next iteration, $\delta$, which is the lesser of $\mu /2$ and 3*h*. The first quantity, $\mu /2$, indicates that we aim to halve the distance to $\mathscr {E}$ in the next simulation by utilizing leaping. The fact that past IRE values are used to construct interpolants instead of past counter data indicates that the system is not producing trajectories that observe $\mathscr {E}$ under the current parametrization. Therefore, setting the next target to $\mathscr {E}$ would be too aggressive and likely result in extrapolation beyond what the data can reliably predict. The second quantity 3*h* sets a maximum limit on the target progress to three times the size of current progress. This limit also ensures extrapolation is not too extreme using the absolute distance to the rare event. If the current state is far from $\mathscr {E}$, halfway point between the latest IRE and $\mathscr {E}$ still may be too far for an accurate extrapolation. By imposing these two limits, we compute $\xi$ more conservatively with IREs than with counters data to account for lack of trajectories reaching the rare event. Pseudo-algorithm for polynomial leaping method is shown in Algorithm 3.
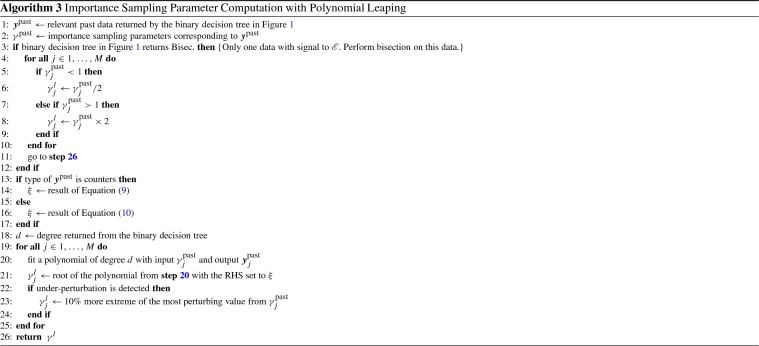


We note that extrapolation of biasing parameters with leaping method can be selectively applied for large systems, where only few reactions may play an important role in observing the rare event. Second example in Sect. 3 illustrates this point.

## Results

In this section, we illustrate the performance of dwSSA$$^{++}$$ by comparing it to that of dwSSA on two example systems—a susceptible–infectious–recovered–susceptible (SIRS) disease dynamics model and a yeast polarization model. In order to minimize the difference in results due to stochasticity, same random number seeds were used for the corresponding dwSSA and dwSSA$$^{++}$$ simulations. Default parameterizations are used for dwSSA$$^{++}$$-specific parameters, i.e., $$l_d = 3 $$ and $$\sigma = 5$$. We emphasize again that the two algorithms differ only in the method for computing optimal biasing parameters, i.e., conventional multilevel CE method vs modified multilevel CE method with polynomial leaping. Once $${\hat{\gamma }^*}$$ is computed, both dwSSA and dwSSA$$^{++}$$ run Algorithm 1 to estimate the rare event probability. All simulations were run using MATLAB$$^{\tiny {\textregistered }}$$ 2017a and Parallel Computing Toolbox™ on Intel$$^{\tiny {\textregistered }}$$ Core™ i7-6400U CPU. All codes used in simulations are available at https://github.com/minroh/dwSSA_pp.

### SIRS

Our first example is a susceptible–infectious–recovered–susceptible (SIRS) disease transmission model, which consists of the following three reactions:

$$\begin{aligned}&R_1: S + I {\mathop {\rightarrow }\limits ^{\beta }} 2I,&\beta&= 0.0675 \\&R_2: I {\mathop {\rightarrow }\limits ^{\lambda }} R,&\lambda&= 3.959\\&R_3: R {\mathop {\rightarrow }\limits ^{\omega }} S,&\omega&= 2.369,\\ \end{aligned}$$

with $\mathbf {x}_0 = [100 \; 1 \; 0]$, where $\mathbf {x} = [S \; I\; R]$. In this model a susceptible individual in *S* becomes infected by an infectious individual in *I* at rate $\beta$. Infectious individuals recover at rate $\lambda$. However, the immunity wanes and the members of *R* rejoin the susceptible pool *S* at rate $\omega$. For this system we examine the event probability $p(\mathbf {x}_0, \theta ^I; t_f) \equiv p([100 \; 1 \; 0], 60;30)$, i.e., probability that the population of *I* reaches 60 before $t_f=30$ given $\mathbf {x}_0$ and $k_0 = [\beta \; \lambda \; \omega ]$. Although the population of all three species stay small throughout the simulation, this particular parameter combination causes the system to exhibit low stochasticity, and the multilevel CE method of dwSSA does not converge by iteration 20 when default simulation parameter values ($\rho = 0.01, K = 10^5$) are used. The most extreme IRE observed in this simulation is 45.

There are two algorithmic parameters—$\rho$ and *K*—that can be tuned to improve speed of convergence albeit each having an associated drawback. The first parameter $\rho$ indicates the fraction of trajectories used to determine an intermediate rare event. Lowering the value of $\rho$ will likely result in an IRE closer to the rare event. However, this also lowers the number of data used to compute the corresponding biasing parameters. Biasing parameters computed with only few data may not be reliable and yield an estimate with high variance. This drawback can be mitigated by increasing the total simulation size *K*. A big disadvantage of increasing the value of *K* for most systems is longer simulation time. However, doing so could lead to convergence for some systems that do not converge with a smaller *K*. Precise relationship between convergence rate and the two parameters is system dependent and often difficult to gauge when nonlinear reactions, such as $R_2$ in the SIRS model, are present.

In order to study the sensitivity of these two parameters in the SIRS model, we computed ${\hat{\gamma }^*}$ using both the dwSSA and dwSSA$^{++}$ with $\rho \in \{10^{-4}, 5\times 10^{-4}, 0.001, 0.005, 0.01\}$ for $K = 10^5$ and $\rho \in \{10^{-5}, 5\times 10^{-5}, 10^{-4}, 5\times 10^{-4}, 0.001, 0.005, 0.01\}$ for $K = 10^6$. We set the maximum number of iterations ($s_{\max }$ in Algorithm 2) to 20 in order to avoid running simulations ad infinitum. If either method did not compute the final biasing parameters by iteration 20, we declared the simulation to be inconvergent. For each run we measured the total simulation time and used it to calculate computational gain of using Algorithm 2 over the conventional multilevel CE method, where $\text {Gain} {:}{=}t(\text {dwSSA})/t(\text {dwSSA}^{++})$. Results from this parameter sweep is summarized in Table 1.

**Table 1:** Results of multilevel CE method and Algorithm 2 applied to the SIRS model

*K*	$\rho$	No. iter.	Convergence	$\hat{\gamma }^{*}$	Tot. time (hr)	Gain $\left( \frac{(\text {dwSSA})}{(\text {dwSSA}^{++})}\right)$
$10^5$	0.01	20	No (45)	NA	1.97	$\infty$
		11 (4)	Yes	(1.222 0.688 1.122)	1.22	
	0.005	20	No (47)	NA	2.10	$\infty$
		7 (2)	Yes	(1.231 0.728 1.130)	0.83	
	0.001	9	Yes	(1.350 0.597 1.230)	1.20	2.27
		5 (1)	Yes	(1.265 0.680 1.116)	0.53	
	$5\times 10^{-4}$	7	Yes	(1.445 0.525 1.234)	0.78	1.03
		7 (2)	Yes	(1.342 0.611 1.293 )	0.76	
	$10^{-4}$	5	Yes	(1.356 0.588 1.318)	0.59	1.26*
		4	Yes	(1.197 0.793 1.264 )	0.47	
$10^6$	0.01	20	No (45)	NA	18.92	$\infty$
		8 (3)	Yes	(1.256 0.666 1.207)	8.09	
	0.005	20	No (46)	NA	19.43	$\infty$
		12 (3)	Yes	(1.369 0.573 1.250)	16.50	
	0.001	20	No (50)	NA	21.90	$\infty$
		7 (2)	Yes	(1.175 0.757 1.051)	7.58	
	$5\times 10^{-4}$	15	Yes	(1.397 0.641 1.174)	19.40	2.19
		7 (2)	Yes	(1.310 0.605 1.222)	8.87	
	$10^{-4}$	5	Yes	(1.146 0.810 1.050)	6.12	1.19
		5(1)	Yes	(1.460 0.586 1.257)	5.14	
	$5\times 10^{-5}$	5	Yes	(1.176 0.816 1.030)	5.60	1.16*
		4	Yes	(1.191 0.747 1.090)	4.82	
	$10^{-5}$	4	Yes	(1.247 0.661 1.251)	4.84	1.07*
		4	Yes	(1.341 0.571 1.097)	4.52	

The first column denotes the ensemble size *K* in multilevel CE method and Algorithm 2, the second column specifies $\rho$, the third column lists the total number of iterations required to compute $\hat{\gamma }^{*}$ if converged (maximum iteration of 20 if not converged), the forth column denotes the observation of rare event where a value in parenthesis indicates the IRE closest to the rare event when the simulation did not converge, the fifth column presents the optimal biasing parameters if computed, the sixth column records the total simulation time, and the seventh column displays computational gain by using the dwSSA$^{++}$ over the dwSSA to compute $\hat{\gamma }^{*}$. Results from using *K* and $\rho$ values from the first two columns are split into two rows—dwSSA(top) and dwSSA$^{++}$(bottom). Number inside the parenthesis in the third column for the dwSSA$^{++}$ simulations indicates the number of iterations that utilized polynomial leaping. Computational gain with * denotes simulations where the difference in results is purely due to stochasticity and not algorithmic difference

Several interesting observations can be made from Table 1. First, it is clear that lowering $\rho$ for a given *K* increases the rate of convergence to the rare event, especially for the conventional multilevel CE method simulations. However, the number of data used to compute $\hat{\gamma }^{*}$ decreases too, and this results in high variability in $\hat{\gamma }^{*}$. For example, dwSSA$^{++}$ does not employ polynomial leaping when using $\rho = 10^{-4}$ and $K=10^5$ (6th row in Table 1), making the algorithm equivalent to the conventional multilevel CE method. However, the two simulations yielded $\hat{\gamma }^{*}$ values that are noticeably different, e.g., 26% difference in $\hat{\gamma }^{*}_{2}$. The difference is not due to $R_2$ being insignificant in producing $\theta ^I$ since $\gamma _2$ stays consistently below 1 throughout the simulation. This is because each iteration of multilevel CE method relied on only the top 10 data ($10^5 \times 10^{-4} = 10$) to compute the next IRE and its corresponding biasing parameters. When either $\rho$ or *K* increases, we see that this variability disappears. For dwSSA$^{++}$ runs that employ polynomial leaping, extrapolation leads to deviation from minimizing cross-entropy, and resulting $\hat{\gamma }^{*}$ is expected to differ from the one obtained by using the conventional multilevel CE method. And the difference does not imply better or worse performance. However, $\hat{\gamma }^{*}_{j}$ values obtained from multiple simulations using the same algorithm and parameterization should be consistent given $R_j$ is involved in rare event production.

In order to demonstrate how high variance in $\hat{\gamma }^{*}$ could negatively affect a rare event probability estimate, we compute $\hat{p}([100 \; 1 \; 0],60;30)$ using $\hat{\gamma }^{*}$ obtained by both algorithms with $\rho = 10^{-4}$ and $K = 10^5$. Running Algorithm 1 with $K=10^7$ and $\hat{\gamma }^{*}_{dwSSA} = [1.356 \; 0.588 \; 1.318]$ yields the following estimate with a 95% confidence interval (Gillespie et al. 2009):

11 $$\begin{aligned} \hat{p}_{dwSSA}^5([50 \; 2 \; 0 \; 50 \; 0 \; 0 \; 0],50;20) = 1.36 \times 10^{-8} \pm 0.03 \times 10^{-8}. \end{aligned}$$

Using $\hat{{\gamma }}^*_{dwSSA^{++}} = [1.197 \; 0.793 \; 1.264]$, on the other hand, yields:

12 $$\begin{aligned} \hat{p}_{dwSSA^{++}}^5([50 \; 2 \; 0 \; 50 \; 0 \; 0 \; 0],50;20) = 1.44 \times 10^{-8} \pm 0.08 \times 10^{-8}. \end{aligned}$$

We can see that the uncertainty from latter simulation is almost three times as large as the one from former even though both were obtained using the same method and same parameter values. Poor sampling and insufficient number of data resulted in this discrepancy. If we increase the total sampling rate by using $K=10^6$ but keep the sample size at 10 by decreasing $\rho = 10^{-5}$, the dwSSA$^{++}$ simulation still remains equivalent to the dwSSA simulation as no leaping is triggered. However, since the total sample size is ten times larger than the former simulation, we see more consistency in the rare event estimate:

13 $$\begin{aligned} \hat{p}_{dwSSA}^6([50 \; 2 \; 0 \; 50 \; 0 \; 0 \; 0],50;20) = 1.43 \times 10^{-8} \pm 0.04 \times 10^{-8}, \end{aligned}$$

14 $$\begin{aligned} \hat{p}_{dwSSA^{++}}^6([50 \; 2 \; 0 \; 50 \; 0 \; 0 \; 0],50;20) = 1.37 \times 10^{-8} \pm 0.04 \times 10^{-8}. \end{aligned}$$

The only way to assess the performance of $\hat{\gamma }^{*}$ is by computing a rare event probability estimate with a large number of simulations, which is often orders of magnitude greater than the number of simulations used to compute $\hat{\gamma }^{*}$. The goal of running either the dwSSA or the dwSSA$^{++}$ is to produce a low-variance estimate, and thus it is important to obtain reliable biasing parameters using sufficient number of data and sampling size. Although lowering $\rho$ and *K* results in faster convergence, it is not worth the computational gain if the resulting biasing parameters yield a high variance estimate.

It is worth noting that the conventional multilevel CE method was not able to compute $\hat{\gamma }^{*}$ for five of the twelve runs in this parameter sweep. We see from Table 1 that dwSSA simulations using $\rho \in \{0.005, \; 0.01\}$ for $K = 10^5$ and $\rho \in \{0.001 \; 0.005, \; 0.01\}$ for $K = 10^6$ did not converge within 20 iterations, while all dwSSA$^{++}$ simulations converged by iteration 12. It is also clear from Table 1 that performance of conventional multilevel CE method is sensitive to changes in both $\rho$ and *K*. On the other hand, performance of Algorithm 2 is robust with respect to both parameters and exhibits superior convergence. Furthermore, because Algorithm 2 utilizes leaping only when it detects slow convergence, it reduces to the conventional CE method when enough progress is being made toward the rare event. This is illustrated by a gradual decline in the number of times polynomial leaping is employed with decreasing $\rho$ (Row 3 in Table 1).

**Fig. 2:**
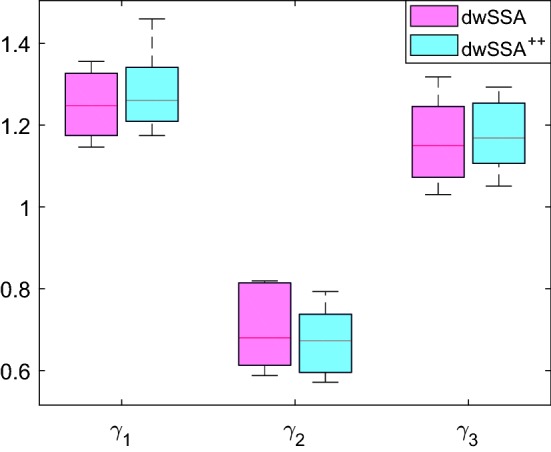
Comparison of ${\hat{\gamma }^*}$ obtained from dwSSA and dwSSA$^{++}$ applied to the SIRS model for $p(\mathbf {x}_0, \theta ^{I}; t_f)$ using $\rho = 0.0001$ and $K=10^5$

Figure 2 displays the spread of biasing parameter values listed in Table 1. We see that $\hat{\gamma }^{*}$ computed by dwSSA$^{++}$ are more consistent in their values and slightly more perturbing than $\hat{\gamma }^{*}$ computed by the dwSSA. We hypothesize that the former phenomenon may be due to dwSSA data having a smaller sample size (7 vs 12 from dwSSA$^{++}$) and the latter due to polynomial leaping, as it pushes the system further than what the multilevel CE method observes.

### Yeast Polarization

For our second example, we use a modified version of the pheromone-induced G-protein cycle in *Saccharomyces cerevisiae*  Drawert et al. (2010) in a similar fashion as Daigle et al. (2011). Our modified system consists of seven species $\mathbf {x} = [R \; L \; RL \; G \; G_a \; G_{bg} \; G_d]$ and is characterized by the following eight reactions:

$$\begin{aligned}&R_1: \emptyset {\mathop {\rightarrow }\limits ^{k_1}} R&k_1&= 3.80 \times 10^{-3} \\&R_2: R {\mathop {\rightarrow }\limits ^{k_2}} \emptyset&k_2&= 4.00 \times 10^{-4} \\&R_3: L + R {\mathop {\rightarrow }\limits ^{k_3}} RL + L&k_3&= 0.084 \\&R_4: RL {\mathop {\rightarrow }\limits ^{k_4}} R&k_4&= 0.0100 \\&R_5: RL+G {\mathop {\rightarrow }\limits ^{k_5}} G_{\alpha } + G_{\beta \gamma }&k_5&= 0.022 \\&R_6: G_{\alpha } {\mathop {\rightarrow }\limits ^{k_6}} G_d&k_6&= 0.100 \\&R_7: G_d+G_{\beta \gamma } {\mathop {\rightarrow }\limits ^{k_7}} G&k_7&= 2.10 \times 10^3 \\&R_8: \emptyset {\mathop {\rightarrow }\limits ^{k_8}} RL&k_8&= 3.21, \\ \end{aligned}$$

with $\mathbf {x}_0 = [300 \; 12\; 0\; 300\; 0\; 0\; 0]$. Past studies showed that the subunit $G_{\beta \gamma }$ plays an important role of signaling for the downstream Cdc42 cycle (McClure et al. 2015; Bar et al. 2003). Here we aim to characterize the probability $p(\mathbf {x}_0, \theta ^{G_{bg}}; t_f)$, where $\theta ^{G_{bg}}$ is defined as the population of $G_{bg}$ reaching 300 and $t_f = 5$.

**Fig. 3:**
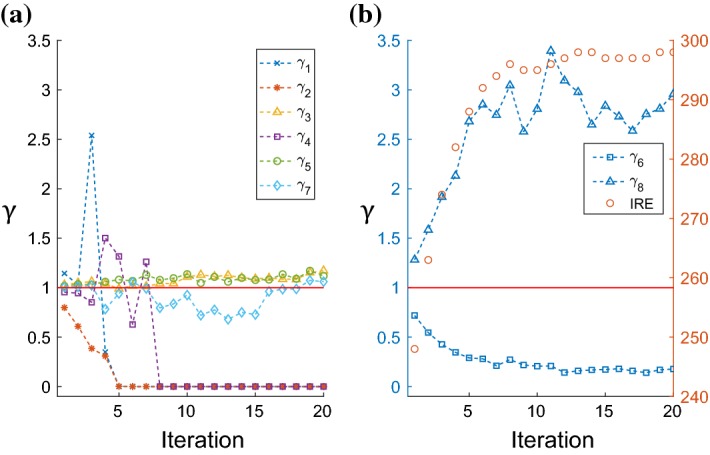
Biasing parameters for the yeast polarization model obtained with the conventional multilevel CE method using $\rho = 0.01$ and $K=10^5$. **a** Displays biasing parameter values for reactions $R_1, R_2, R_3, R_4, R_5,$ and $R_7$, where their values are not consistently different from 1. **b** Displays biasing values for reactions $R_6$ and $R_8$ using the left *y*-axis. These two biasing parameters consistently deviate from 1 throughout 20 iterations. Right *y*-axis is used to display the intermediate rare event corresponding to the iteration specified by the *x*-axis

Daigle et al. (2011) studied this system under a different parameterization. While the reaction rates and the rare event definition differ, their rare event also examines the population of $G_{\beta \gamma }$ ($\theta ^{G_{bg}} = 50$). The authors note that the two reaction rates that are most consistently differed from 1 are $\gamma _6$ and $\gamma _8$, both of which do not directly affect the population of $G_{\beta \gamma }$. This is illustrated in their paper by running 100 independent runs of multilevel cross-entropy method and computing variability among final biasing parameters. They also show that most simulations converge in three iterations. In our example, multilevel CE method with the same simulation parameters ($\rho = 0.01$ and $K=10^5$) does not converge by iteration 20 and no biasing parameters are computed to estimate the rare event probability. However, when analyzing the resulting biasing parameters in each iteration of the multilevel CE method, same trend emerges; only biasing parameters $\gamma _6$ and $\gamma _8$ are consistently and significantly different from 1. Rest of the biasing parameters are either not consistent in the direction of basing or remain close to 1. We show the two different trends in Fig. 3. In Fig. 3a we see that all six parameters either fluctuate above and below 1 ($\gamma _1, \gamma _2, \gamma _4$, and $\gamma _7$) or stays very close to it ($\gamma _3$ and $\gamma _5$). On the other hand, $\gamma _6$ and $\gamma _8$ values in Fig. 3b are consistently and significantly below and above 1, respectively. It is worth pointing out that $\gamma _1$, $\gamma _2,$ and $\gamma _4$ all degenerate to 0 as intermediate rare event gets closer to the rare event. The first two parameters quickly approach 0 by iteration 5, and $\gamma _4$ is “turned off” by iteration 8. We can interpret this as multilevel CE method reducing the system to a lower dimensional model as some reactions become unnecessary in observing the rare event. When there is no observation of a reaction, we set its biasing parameter to the minimum positive number allowed in the computing hardware. This way the reaction is always possible to fire in the next iteration, however unlikely it may be.

As system size grows, it is likely that some, if not most, of the reactions do not affect observation of the rare event. Biasing parameters for reactions that do not matter in rare event production will show high variability in their values and are easy to detect Daigle et al. (2011). When leaping method is utilized, these biasing parameters should be excluded from being extrapolated for computational efficiency. Incorporating these spurious parameters in polynomial leaping will not only increase the total computation time but also produce mathematically meaningless interpolant. Therefore, we apply polynomial leaping on only the two parameters, $\gamma _6$ and $\gamma _8$, that affect observation of the rare event. The rest of the biasing parameters retain their value from the previous iteration as they are deemed unimportant in rare event observation. However, their values are updated when the multilevel CE method (Algorithm 2 step 15) is run instead of the polynomial leaping method. This enables the algorithm to collect data and use them to decide which reactions need to be extrapolated when there is slow or no convergence. Table 2 summarizes simulation results from running both dwSSA and dwSSA$^{++}$ to compute $\hat{\gamma }^{*}$ for $p(\mathbf {x}_0, \theta ^{G_{bg}}; t_f)$ using $K = 10^5$ and $\rho \in \{10^{-4} \; 5\times 10^{-4} \; 0.001 \; 0.005 \; 0.01\}$.

**Table 2:** Results of multilevel CE method and Algorithm 2 applied to the yeast polarization model

*K*	$\rho$	No. iter.	Convergence	$[\hat{\gamma }^*_6 \; \hat{\gamma }^*_8 ]$	Tot. time (hr)	Gain $\left( \frac{(\text {dwSSA})}{(\text {dwSSA}^{++})}\right)$
$10^5$	0.01	20	No (298)	NA	1.61	$\infty$
		10 (2)	Yes	(0.120 4.445)	0.72	
	0.005	16	Yes	(0.112 3.194)	1.20	1.47
		12 (3)	Yes	(0.0889 3.352)	0.82	
	0.001	8	Yes	(0.146 3.174)	0.62	1.20
		7 (1)	Yes	(0.0846 3.062)	0.52	
	$5\times 10^{-4}$	7	Yes	(0.124 3.420)	0.55	1.01*
		7	Yes	(0.130 2.896)	0.55	
	$10^{-4}$	5	Yes	(0.169 2.865)	0.38	0.82*
		6	Yes	(0.176 3.331)	0.47	

Column identities match those of Table 1. Only $\gamma _6$ and $\gamma _8$ are listed in column 5

Similar to the SIR model, we see a gradual increase in performance with decreasing value of $\rho$ when using the conventional multilevel CE method, while the performance of Algorithm 2 is relatively robust with respect to $\rho$. Algorithm 2 converges in all five sets of simulations while the multilevel CE method does in only three. As the convergence rate increases with decreasing $\rho$, leaping method is triggered less frequently, and the two methods eventually become equivalent when no leaping is employed. When there is no leaping, any difference in the performance is purely due to stochasticity. We note that it is possible to modify Algorithm 2 to dynamically choose biasing parameters that can be used for extrapolation when leaping method is triggered. When slow convergence is detected, past biasing parameter values can be scanned to select leaping indices prior to entering Algorithm 3. Therefore, it is not necessary to run simulations prior to decide which reactions are to be extrapolated.

**Fig. 4:**
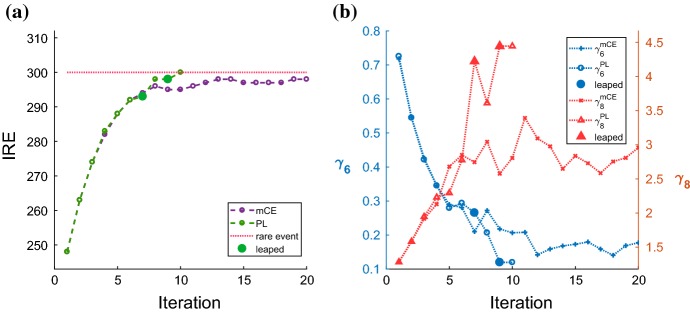
Comparison of results between dwSSA and dwSSA$^{++}$ applied to the yeast polarization model. Both algorithms were run to estimate ${\hat{\gamma }^*}$ for $p(\mathbf {x}_0, \theta ^{G_{bg}}; t_f)$ using $\rho = 0.01$ and $K=10^5$. **a** Displays progression of intermediate rare events by iteration. **b** The two biasing parameters chosen for leaping—$\gamma _6$ and $\gamma _8$ are shown by iteration

We illustrate effectiveness of leaping with $\rho = 0.01$ and $K=10^5$ on Fig. 4. Only the dwSSA$^{++}$ converges using this parameter combination after utilizing leaping twice in iterations 7 and 9. The conventional multilevel CE method gets close to producing the rare event but never reaches it by iteration 20. We see from Fig. 4a that lack of time is not the main cause, as the dwSSA observes $G_{\beta \gamma } > 290$ after iteration 6. The maximum $G_{\beta \gamma }$ population in this simulation is 298, and it is first observed during iteration 13. We hypothesize that the system entered a stochastic equilibrium around this time, and that prevented the algorithm from converging. On the other hand, dwSSA$^{++}$ recognizes slow convergence first at iteration 7 and then again at iteration 9. By extrapolating $\gamma _6$ and $\gamma _8$ values using past IRE data, the algorithm reaches the rare event by iteration 10 and successfully computes $\hat{\gamma }^{*}$. Figure 4b shows $\gamma _6$ and $\gamma _8$ values computed by both algorithms. We see that the most significant change in $\gamma _6$ from dwSSA$^{++}$ occurred during the first leaping and $\gamma _8$ during the second leaping.

## Conclusion and Discussion

This paper describes dwSSA$^{++}$ and its novel contribution in improving automatic computation of biasing parameters required to characterize a rare event probability. Numerical results from two example systems in Sect. 3 support our claim that the polynomial leaping method employed by dwSSA$^{++}$ can significantly shorten simulation time in computing biasing parameters. We showed that the 12 simulations that employed polynomial leaping at least once performed better than its corresponding dwSSA simulations. Furthermore, the dwSSA$^{++}$ converged on all 17 sets while the dwSSA failed to compute biasing parameters on 6 of them. Thus, the benefit of using dwSSA$^{++}$ is not limited to computational efficiency but also lowering the failure rate in computing biasing parameters.

We note that the main contribution of dwSSA is in automatic computation of biasing parameters. Similar methods existed prior to dwSSA that utilized importance sampling to efficiently estimate a rare event probability (Kuwahara and Mura 2008; Gillespie et al. 2009; Roh et al. 2010), but they were all impractical for large systems because there was no principled method to compute biasing parameters that could yield a low-variance probability estimate. Therefore, the dwSSA is as impractical as its predecessors without automatic computation of the biasing parameters. Although the multilevel CE method used in dwSSA works well for many systems, it can fail to converge when a system is in a stochastic equilibrium or exhibiting low stochasticity. The dwSSA$^{++}$ attempts to resolve this problem by extrapolating biasing parameters using past simulation data when slow convergence is detected. The algorithm also offers tuning parameters that define the threshold for slow convergence and the amount of past data utilized in polynomial leaping method. This allows for flexible controlling of the algorithm.

We point out that simulations run with the default parameter values ($\rho = 0.01$ and $K = 10^5$) described in Daigle et al. (2011) are inconvergent for both examples shown in this paper. However, we note that these values work well for many systems (Daigle et al. 2011; Roh et al. 2011) that do not suffer from low stochasticity. Default parameter values $\rho = 0.01$ and $K=10^5$ ensure both sampling frequency ($10^5$) and the number of data used to compute IREs ($10^5 \times 0.01 = 10^3$) are adequate. Thus, unless a user is aware that the system under study exhibits low stochasticity prior to simulation, we suggest the user to run the dwSSA$^{++}$ with default parameter values and allow the algorithm to extrapolate the biasing parameters when necessary. As shown with examples in Sect. 3, dwSSA$^{++}$ adaptively switches between the polynomial leaping method and the multilevel CE method depending on the system behavior.
